# Estimating the impact of the COVID-19 pandemic on dengue in Brazil

**DOI:** 10.21203/rs.3.rs-2548491/v1

**Published:** 2023-02-09

**Authors:** K. O. Roster, T. Martinelli, C. Connaughton, M. Santillana, F. A. Rodrigues

**Affiliations:** 1 Institute of Mathematics and Computer Science, University of São Paulo, São Carlos, SP, Brazil; 2 Mathematics Institute, University of Warwick, Coventry, United Kingdom; 3 London Mathematical Laboratory, London, United Kingdom; 4 Machine Intelligence Group for the Betterment of Health and the Environment, Network Science Institute, Northeastern University, Boston, MA, USA; 5 Center for Communicable Disease Dynamics, Harvard T.H. Chan School of Public Health, Boston, MA, USA

## Abstract

Atypical dengue prevalence was observed in 2020 in many dengue-endemic countries, including Brazil. Evidence suggests that the pandemic disrupted not only dengue dynamics due to changes in mobility patterns, but also several aspects of dengue surveillance, such as care seeking behavior, care availability, and monitoring systems. However, we lack a clear understanding of the overall impact on dengue in different parts of the country as well as the role of individual causal drivers. In this study, we estimated the gap between expected and observed dengue cases in 2020 using an interrupted time series design with forecasts from a neural network and a structural Bayesian time series model. We also decomposed the gap into the impacts of climate conditions, pandemic-induced changes in reporting, human susceptibility, and human mobility. We find that there is considerable variation across the country in both overall pandemic impact on dengue and the relative importance of individual drivers. Increased understanding of the causal mechanisms driving the 2020 dengue season helps mitigate some of the data gaps caused by the COVID-19 pandemic and is critical to developing effective public health interventions to control dengue in the future.

## 1. Introduction

Measures to curb the spread of SARS-CoV-2 and shifts in resource allocation during the COVID-19 pandemic have impacted the dynamics and surveillance of dengue, causing data gaps on the true disease burden, especially in 2020. Given the geographic diversity of dengue seasonality, timing, and intensity, we expect that different countries and regions will have experienced distinct impacts due to the initial non-pharmaceutical interventions conceived to curb the effects of the COVID-19 pandemic in 2020.

In most dengue-endemic countries in Asia and Latin America, the COVID-19 pandemic reduced the observed dengue activity in 2020 by an estimated 0.72 million cases [13], resulting in a reduced pooled relative risk of infection of 0.55 when compared to pre-pandemic historical trends[50]. Brazil overall deviates from this trend, because it experienced more dengue cases than expected [13], leading to a local relative risk ratio of 13.25 in 2020 compared to a pre-pandemic period from 2014 to 2019 [50]. With temporal and geographical exceptions, such as those documented by Conceição et al. [15], who found that risk of dengue infection in the Brazilian state of São Paulo decreased by 9 percent twenty days after the start of social isolation.

Brazil is a very large country with diverse environmental and climatic conditions, resulting in varying levels of dengue incidence and endemicity. To better understand the impacts of the COVID-19 pandemic on dengue activity in Brazil, it is therefore important to study this at sub-national geographic scales. Existing work analyzing the impact of the COVID-19 pandemic on observed dengue in Brazil has attributed changes in observed dengue activity to changes in human mobility and NPIs alone [13, 15, 50], without quantitatively assessing the contribution of the other known causal drivers of dengue dynamics, including the altered dengue surveillance capabilities during the pandemic.

Previous research in Brazil has characterized the wave-like pattern of dengue spread across the country moving from western to eastern regions, which may be explained by distinct climate seasons, specifically rainfall patterns, and to a lesser extent by human movement [14]. Castro et al (2021) documented geographic differences in the intensity and seasonality of outbreaks, as well as the pairwise associations between outbreak timing and disease trends across locations. These geographic differences in annual dengue behavior may help better characterize the mechanisms behind the impacts of the COVID-19 pandemic on dengue activity, since they allow us to analyze different relative timings of the onset of COVID-19 activity in relation to the local dengue seasonality across the country [11].

### Role of Surveillance Systems.

Observed dengue cases are a consequence of not only disease dynamics but also depend on the ascertainment capacity of the associated surveillance systems. Ascertainment of a dengue infection relies on the (i) care-seeking behavior of the patient–which may depend on the trust of people on the health care system–, (ii) availability of care, and (iii) subsequent reporting upon diagnosis. All three of these factors may have been dramatically affected by the pandemic. In fact, research shows that patients were less likely to seek care at the start of the pandemic [46, 16, 2], and overburdened healthcare systems had a lower capacity to treat other conditions, and complete the sometimes time-consuming reporting procedures [27].

The Brazilian National System for Surveillance and Control of Diseases (SNVS) is integrated in a universal healthcare system and supported by an open data initiative. The strength of infectious disease surveillance is demonstrated by the successful control of vaccine-preventable diseases such as tetanus and pertussis [5]. The significant reduction of the vector-borne Chagas disease also exemplifies Brazil’s strong history of effective vector control programs [39, 5]. Yet, the pandemic challenged surveillance systems of various diseases in many countries [16, 2, 35], including Brazil [46, 6], likely leading to a lower case ascertainment rate in 2020.

Even before the pandemic, under-reporting of dengue was a known challenge. An estimated 84 percent of dengue infections are asymptomatic [45]. In a serological study in a healthcare facility in the city of Salvador, Brazil, Silva and colleagues (2016) found that the reporting system captured only 5 percent of patients with dengue who sought care. [43]. A complete notification of a suspected dengue case involves filling 71 items in a digital or paper form, which can become an obstacle to reporting dengue cases due to the time-consuming nature of the process –even in the absence of the increased burden on healthcare systems during the COVID-19 pandemic [3].

Not only excess under-reporting may have affected dengue surveillance, but a competing factor may have led to over-reporting of dengue cases in the presence of other infections, including a misdiagnosed COVID-19 infection. Even before the COVID-19 pandemic, the false positive rate of reported cases was 31 percent in high-transmission periods and 62 percent in low-transmission periods [43]. Since dengue is a nationally notifiable disease, healthcare providers are required to report suspected cases for patients showing fever and at least two of the following symptoms: headache, retro-orbital pain, myalgia, arthralgia, exanthema, bleeding or hemorrhage, nausea or vomiting, petechiae or positive tourniquet test (rash), or leukopenia (low white blood cell count) [33, 43] in an area of dengue transmission or *Aedes aegypti* infestation presents. These symptoms overlap with common symptoms of COVID-19. Additionally, cross-reactivity of laboratory tests for COVID-19 and dengue has been reported [38, 32, 29].

After accounting for changes in ascertainment and reporting, several variables remain as candidates to explain any atypical dengue case counts (see also the causal graphical model described in Supplemental Section 5), including human mobility, climate conditions, and human susceptibility, described in more detail below.

### Role of human mobility.

Given the short travel radius of *Aedes mosquitos* of 100–400 meters [49, 34, 31], human mobility contributes to the introduction and spread of dengue at multiple geospatial scales. For example, transport networks explain some of the variability in dengue cases in the city of Bangkok, Thailand [28]. Mobile phone data improves predictions in Thai provinces [26] and across Pakistan [47]. In Brazil, human connectivity helps explain the correlation of dengue outbreak timing and trend between different mesoregions [11]. Churakov et al. (2019) find rainfall to be more predictive of dengue seasonality than mobility (as approximated by a gravity model) across mesoregions and finer geospatial scales, though the gravity model was informative at higher aggregations [14].

The mechanisms through which mobility impacts dengue dynamics are increasingly understood and allow for the possibility of both positive and negative effects on dengue prevalence during the pandemic. Human mobility can be the driving force of both the outbreak size and the speed of spread across neighborhoods [4]. Specifically, the variability of mobility supports dengue spread, while the time spent in a given place has little impact [1]. Yet other factors, such as mosquito density and biting suitability of infected individuals are also strong determinants of whether mobility reductions (in response to symptomatic infection) lead to increases or decreases in dengue transmission [41]. This suggests that mobility changes due to the pandemic may have also impacted dengue dynamics in significant, though possibly geographically diverse ways.

### Role of mosquito prevalence and climate variables.

Dengue transmission in humans is mediated by *Aedes aegypti* and *Aedes albopictus* mosquitoes, whose population size is affected by environmental (e.g. presence of larval breeding sites) and climate conditions (e.g. temperature, humidity, and precipitation). These variables define the limits of vector survival, within which we distinguish between viable and optimal conditions. Campbell and colleagues (2013), for example, find that 80 percent of dengue cases in Thailand occurred when mean humidity was above 75 percent and mean temperature was within 27–29.5°C [10]. This is because the extrinsic incubation period decreases at warmer temperatures, thus reducing the time it takes for a mosquito to transmit the virus to humans [12]. Similarly, rainfall and flooding can provide additional breeding habitats for Aedes mosquitoes, for example after extreme weather events [18, 17]. Dengue is observed in all states in Brazil, though climate zones span tropical and sub-tropical regions, thus varying the timing and duration of viable conditions.

Upon contact with an infectious mosquito, the probability of human infection is determined by a person’s immune status. Previous infection with a dengue serotype conveys lifelong immunity against that serotype and temporary, partial immunity against other serotypes [49]. Human susceptibility to dengue may thus be reduced after large outbreaks, as was the case after the Zika epidemic in 2016 [8].

Dengue dynamics are therefore driven by a complex interaction of biological, ecological, and social factors of the vector, host, and pathogen, making it difficult to separate the effect of the pandemic from other dengue drivers. We currently lack complete knowledge of the causal drivers of dengue dynamics generally, and specifically of the active drivers during the COVID-19 pandemic as well as their relative importance in different parts of the country.

### Our contribution.

In this study, we aim to understand how shifts in human behaviour and other likely causal drivers, such as climate variables, impacted dengue activity in Brazil during the early stages of the COVID-19 pandemic. We allow for variability of pandemic effects across states and analyze the role of each causal driver in a hierarchical approach. First, we estimate the impact of the pandemic as a whole on dengue in Brazil at different geographical aggregations using an interrupted time series causal inference design. Next, we investigate in turn whether the gap between expected and observed dengue can be attributed to pandemic-related factors or other known covariates of dengue activity, specifically climate conditions, human mobility, human susceptibility, and adjustments for pandemic-related changes in surveillance.

These estimates of the overall impact of the COVID-19 pandemic on dengue in different parts of Brazil may help mitigate some of the data gaps from 2020 and support forecasts of dengue in the post-pandemic period. Our work may also contribute to an increased understanding of the causal drivers of dengue dynamics more generally, which is critical to developing effective public health interventions.

## 2. Materials and Methods

### 2.1. Data

We use weekly observed dengue case counts from the Brazilian Notifiable Diseases Information System (Sistema de Informação de Agravos de Notificação - SINAN) from 2014–2020 [21]. Climate data are extracted from the Brazilian National Institute of Meteorology (Instituto Nacional de Meteorologia - INMET) over the same time period and include variables on rainfall, humidity, temperature (max, min, median) and wind speed [20]. Changes in human mobility are assessed using Google Regional Mobility Reports, which capture relative changes in time spent in residential areas, transit stations, and other points of interest in cities across Brazil during the pandemic relative to a pre-pandemic baseline. We supplement this empirical mobility data with information on non-pharmaceutical interventions implemented in different states from the Oxford Coronavirus Government Response Tracer database [23]. To approximate changes in care-seeking behavior and care availability, we collect data on treatments for conditions related to an HIV infection (ICD-10 codes B20–24 and Z21) and elective hospital internations from the Brazilian Ministry of Health (DATASUS) [40]. SARS-CoV-2 infections across municipalities are reported by the states’ Secretariat of Health and consolidated at *Brasil.io* [25].

**Figure 1:**
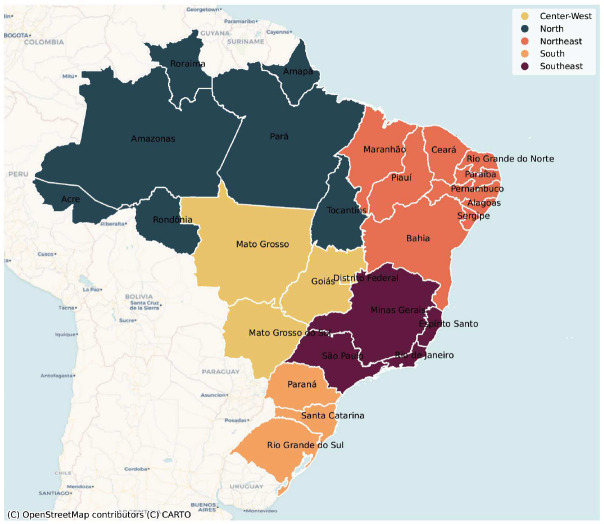
Study area

For all datasets, we consider several levels of geospatial detail, including the five regions of Brazil, 27 Federative Units (which include all states and the capital city, Distrito Federal), 136 mesoregions, and 5570 municipalities (Figure 1).

### 2.2. Outbreak characteristics

In addition to case counts, we also consider the shape of annual dengue time series. Leveraging methods previously described in [11], we compute measures for the intensity and seasonality of the dengue season. The intensity measures how concentrated dengue cases are in specific weeks of the year. The seasonality measures how regular outbreaks occur in 52-week intervals.

### 2.3. Outlier Analysis

We consider deviations from typical behavior along several dimensions: the dengue case count, the shape of the dengue outbreak (intensity, seasonality, peak, and onset), and climate variables. To compute outliers, we first transform the data to near-Gaussian distributions using a Box-Cox or Yeo-Johnson transforms, depending on fit. We then compute z-scores for all observations based on distributions for each epidemiological week in each state over the pre-pandemic period (2014–19). All observations in 2020 with z-scores greater than 2.58 (corresponding to *p* < 0.01 in a two-sided t-test) are considered outliers. In a sensitivity analysis, we also compute outliers according to the thresholds *p* < 0.05 and *p* < 0.1. For the weekly variables, we analyze the share of all week-city combinations that are outliers. The intensity and seasonality are annual measures, therefore they are compared only across cities, not weeks.

### 2.4. Interrupted Time Series Analysis

Interrupted time series analysis (ITSA) leverages sudden disruptions to data generation mechanisms to estimate causal impacts of interventions from time series data [42, 30]. A predictive model is fit to training data up to the implementation time of the intervention. Inputs may consist only of the outcome variable or also include relevant covariates that help model the trend or seasonality of the time series. The observed outcome is then compared to predictions of post-intervention observations to understand how the level and trend of the time series changed in response to the intervention. Interventions may be implemented as part of an experiment designed by a researcher to estimate a specific causal effect or they may be the result of a natural experiment, which occurs without interference by the researcher [42], as is the case in this study.

ITSA is commonly implemented with models of the autoregressive integrated moving average (ARIMA) family and recently with the more general set of Bayesian structural time series (BSTS) models. The advantage of these models is their ability to explicitly handle different time series components, such as the trend and seasonality. Green and colleagues (2021), for example, implemented an interrupted time series design with ARIMA, BSTS, and negative binomial regression models to estimate the impact of the COVID-19 lockdown announcement in the UK on misinformation shared on Twitter [22]. BSTS-based ITSA has also been applied to study the impact of reforms in drug monitoring policies on mortality rates [19]. The use of machine learning models for quasi-experimental causal inference designs in general, and ITSA specifically is comparatively underexplored [7].

In this study, we implement an ensemble consisting of a feed-forward neural network (NN) and a BSTS model in an interrupted time series design. The BSTS model is defined as described in [9] with a Gamma prior distribution. After hyperparameter selection, the NN model is constructed with two hidden layers of 128 units each, rectified linear unit activation, Adam optimization, and a learning rate of 0.001. We apply this ITSA approach in two ways. First, we compare the expected dengue cases (the output of our ITSA approach) with those that were observed to understand where the 2020 dengue season was atypical. In this analysis, we consider different interruption times to assess the two main interventions of interest, mobility reductions and surveillance disruptions. The first time point was selected to be March 7th 2020, the point in time when the largest changes of human mobility were observedon empirical mobility. To quantify disruptions to dengue surveillance due to COVID-19, we selected January 13th, 2020, the point in time when the first case of COVID-19 was reported outside of Wuhan, [48], and consider all other dates up to March 14th as part of a sensitivity analysis. These dates allow us to test different hypotheses, based on the assumption that the data collection mechanism was unaffected until the interruption point, and that any changes in the dengue time series were caused by pandemic interventions on those dates. Two states, Amapá and Roraima, are excluded from the ITSA analysis, because of missing climate observations during part of 2020.

### 2.5. Reporting adjustment

The second implementation of ITSA is applied to estimate the pandemic-induced excess under-reporting. We compare expected and observed quantities of HIV-related treatments and elective hospitalizations, to approximate reductions of care-seeking bahaviors and health care availability, respectively. The percentage change in these two proxy time series are then used to compute *adjusted dengue cases*, which may be interpreted as the dengue cases that would have been observed in the absence of changes in care seeking and care availability, respectively. Specifically, we compute reporting-adjusted dengue cases $d_{t}^{*}=d_{t}\left(1-u_{t}\right)$, with the percentage change in reporting *u* determined by $u=\frac{a_{t}-\hat{a_{t}}}{a_{t}}$, where *d*_*t*_ are observed dengue cases at time *t*, *a*_*t*_ is the observed quantity of the reporting approximation, and $\hat{a_{t}}$ is the expected quantity of the reporting approximation predicted by ITSA.

## 3. Results

### 3.1. Is the 2020 observed dengue season unusual?

#### North.

**Figure 2:**
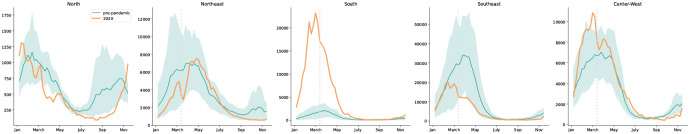
Dengue time series by epidemiological week in 2020 and in the pre-pandemic period 2014–19, by region Green lines and shaded regions represent the mean and 95% confidence interval of dengue cases in regions computed for each epidemiological week over the years 2014 to 2019. Orange lines represent the 2020 dengue case count each week. Gray dotted lines indicate the start of the COVID-19 pandemic in 2020.

**Figure 3:**
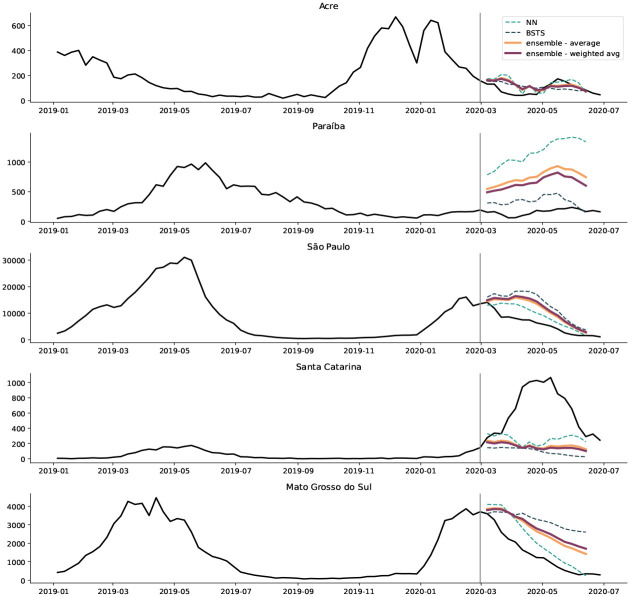
Observed and expected dengue case counts in five sample states, at interruption time 29 February 2020 Observed dengue cases (black) are compared to 15-week ahead forecasts of the feed forward neural network (NN), the bayesian structural time series model (BSTS), and a simple average as well as a weighted average ensemble of the two individual forecasts.

The North of Brazil has the earliest start of the annual dengue season and maintains low levels of active infections throughout the year due to its tropical climate [14] (Figures 2 and 11). The pandemic is expected to have less of a cumulative impact on dengue in this region, since mobility reductions and surveillance changes occurred toward the end of the dengue season in these states. At the regional level, ITSA suggests that observed cases closely follow the expected trajectory, though forecasts at the state-level are more volatile (Figures 3 and 13). The states Acre, Amazonas, and Rondônia experience some dengue anomalies at the city-level (6), though these tend to occur before and at the end of the first pandemic wave, in early January and in June.

#### Northeast.

According to the ITSA results, states in the Northeast generally experienced slightly fewer cases then expected, with an especially pronounced temporary dip in cases in the two weeks following March 14th.

Ceará, for example, begins the season with higher-than-expected cases, followed by a dip in reported cases in mid-March.

Bahia deviates from the other states in the Northeast. Its outbreak is larger than expected, regardless of the time series interruption point, though it is not unprecedented: case counts are almost as large as in the 2016 outbreak and the outbreak timing is comparable to the 2019 season.

#### Southeast.

The Southeast region also observed fewer dengue infections than expected. In Minas Gerais and Rio de Janeiro, cases are below the ITSA predicted values for all interruption times. In São Paulo, the season starts with higher-than-usual incidence, but cases begin to decline at the end of February. The government of Espírito Santo stopped using the online disease notification system in 2020 [21], leading to clearly evidenced and sudden-onset underreporting of dengue cases (Figures 2 and 11), which helps us validate the ITSA results. Both the individual and ensemble ITSA forecasts recognize this break in the data generation mechanism and identify the influence of under-reporting, by predicting higher cases than reported (see Supplement).

#### South and Center-West.

In states in the South and Center-West, ITSA results are similar. In most states, cases are higher than expected for early interruption times. If we assume that the observed cases reflect the true disease dynamics, ITSA results shift to lower than expected case counts for later interruption times. In other words, the pandemic impacts either dampened an outbreak that would have been even larger or there were changes in early disease drivers that led to increased cases. There are two exceptions in these regions. In Santa Catrina (South), dengue is greater than expected for all interruption times. In Goiás (Center-West), the dengue time series is as expected for all interruption points, except for a dip in reporting around March 7th. This dip is also observed in the other states in the region.

### 3.2. Role of excess under-reporting

Dengue surveillance is influenced by people’s care-seeking behavior, health care availability, and reporting capacity. Using the approximations for changes in the former two of these factors, we explore whether and to what extent lower-than-usual dengue cases could be explained by changes in surveillance systems, specifically by excess under-reporting during the pandemic. We assume that under-reporting of dengue cases, while common, was somewhat consistent in time before the COVID-19 pandemic and then that it may have increased during the pandemic. Using a separate ITSA on the proxy datasets (as described in section 2) We compute “adjusted dengue cases”, which approximate the level of observed dengue cases we would have expected without excess under-reporting in the period from March to June of 2020. This measure is different from the expected dengue cases that was computed in the previous section, since it accounts only for excess under-reporting, not the other pandemic or non-pandemic drivers of dengue dynamics. The adjusted time series therefore represents the observed cases that would have been expected under pre-pandemic under-reporting conditions.

**Figure 4:**
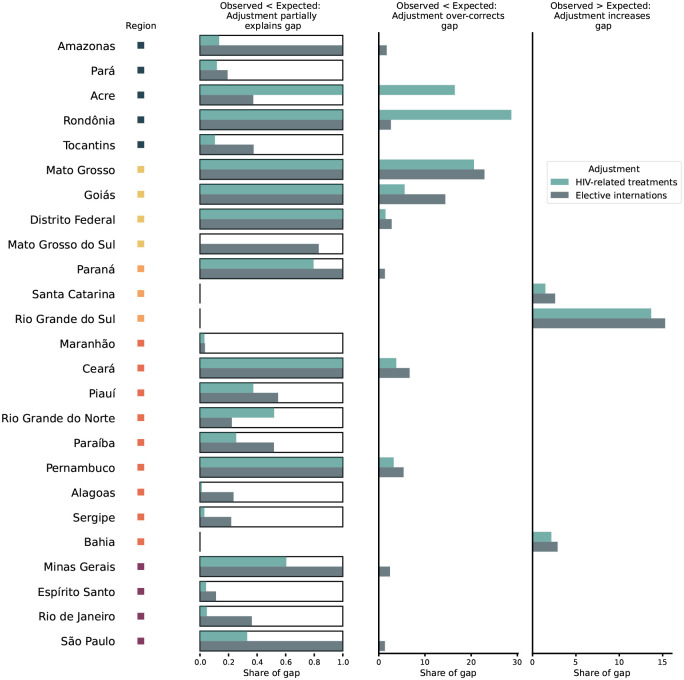
Summary of adjustment for reporting changes as approximated by HIV-related treatments and elective internations The figure compares the cumulative gap between expected and reported cases from March to June 2020 with the gap between adjusted and expected cases. Adjustments are computed using time series of HIV-related treatments (light green) and elective internations (dark green). Each of the three columns represents a different category of adjustment results: The first column shows states, where observed cases are lower than expected cases, and at least one of the two adjustments is within the observed-expected gap (Observed < adjusted < expected). Column two shows cases where the adjustment over-corrects for the gap, such that the adjustment is greater than the prediction (Observed < expected < adjusted). Finally, the third column shows the cases where observed cases are greater than expected cases, and adjustment further increases the gap. All x-values are expressed as shares of the observed-expected gap.

Of the 25 states in the ITSA analysis, 22 reported fewer dengue cases than expected for the March 1st interruption time point (Figure 4). In 15 of these states, the gap is partially explained by at least one of the two adjustments. In 11 states, adjustment over-corrects for the gap between observed and expected cases for at least one of the two approximations, so that adjusted cases are higher than expected cases. The remaining 3 of 25 states report more dengue cases than predicted and adjustment (according to both approximations) further widens the gap between observed and expected cases.

#### North.

In the North, the adjustment results in a mix of partial compensation and over-correction (Figure 4).

#### Northeast.

In most of the Northeastern states, adjustment for excess under-reporting partially explains the gap between expected and observed cases. In Ceará and Pernambuco, the adjustment over-corrects for the gap. Bahia is an outlier in the region, since it observed more cases than expected and adjustment further increases the gap (Figure 4).

#### Southeast.

**Figure 5:**
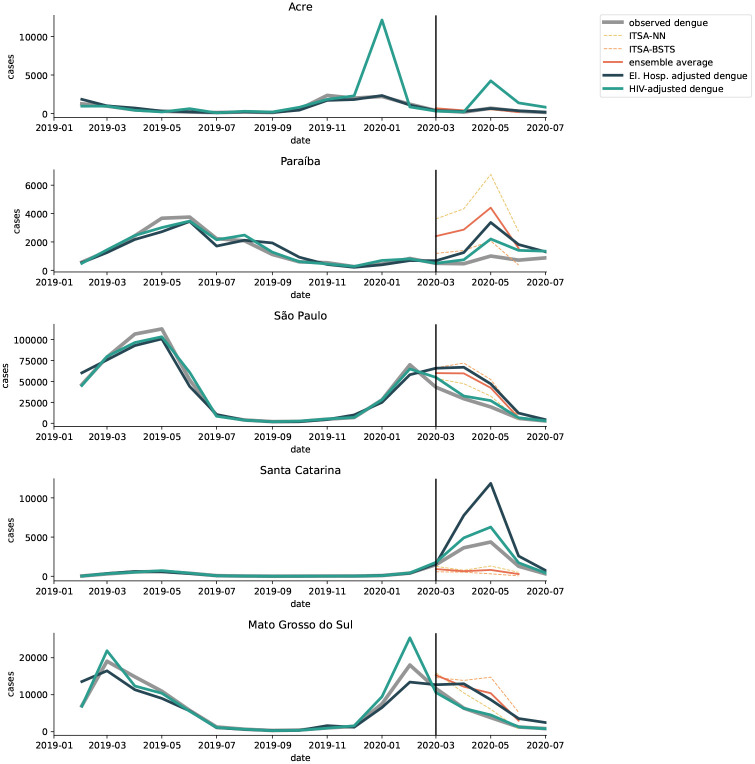
Dengue cases adjusted for excess under-reporting during the pandemic, compared with expected and observed dengue cases in five sample states

In all four states in the Southeast, adjustment reduces the gap between observed and expected cases for at least one of the two proxies (Figure 4). In São Paulo, for example, we note that the adjustment using elective surgeries as a proxy for reduced care availability, very closely follows the expected dengue cases (Figure 5). Thus, reduced case ascertainment due to overwhelmed hospital systems may explain the low case count observed in São Paulo during the pandemic.

#### South.

As shown in the ITSA, cases in the southern region were atypically high in 2020. We also observe evidence for lower case ascertainment due to lower care-seeking behavior and care availability, as measured by the two proxy time series. Adjusting for this excess under-reporting further widens the gap between expected and observed cases (Figures 4 and 5). In the absence of other influences, such as over-reporting or mobility, the outbreak in this region may have been even larger than what was observed.

#### Center-West.

While the outbreak in many states in the Center-West were larger than expected, the ITSA for later interruption points (assuming no structural changes before March 2020) showed observed cases to be slightly above expected levels. Therefore, adjusting observed cases upwards further increases the gap in Mato Grosso, Goiás, and Distríto Federal (Figure 4). This could indicate that the above-average dengue outbreak would have continued in the absence of the COVID-19 pandemic, and fewer cases were ascertained at the end of the dengue season. In Mato Grosso do Sul, adjusted dengue cases (using the elective surgery proxy) are comparable to expected cases, which are greater than observed cases for the March 1st interruption point (Figure 5).

### 3.3. Role of climate conditions

We compute the outliers (*p* < 0.01) of the climate variables for the first half of 2020. We find that 6 percent of week-city combinations are outliers on the distribution of rainfall and 4.5 percent on the distribution of humidity. The other climate variables had lower outlier rates.

#### North.

The two most northern states, Roraima and Amapá, experienced lower humidity in the first quarter. The two most southern states in the Northern region, Acre and Rondônia, had elevated minimum temperatures, meaning warmer nights. The state of Amazonas had a lower daily temperature range, with higher median temperatures and lower maximum temperatures. Pará’s humidity outliers occured late into the year after the dengue season and pandemic interventions. Tocantins experienced slightly elevated precipitation in the first quarter.

#### Northeast and Southeast.

Bahia experienced weeks with strong rains, which could be a contributing factor for the unusally high dengue case counts. Increased rainfall is linked with greater dengue incidence, as it helps create larval breeding sites for mosquitoes, though too much rain may wash away existing breeding sites, which may explain why Minas Gerais did not experience increased dengue incidence as a result of the similarly strong rains.

In the remaining states in the Northeast and Southeast, climate conditions were closer to typical years, but with some outliers in rainfall and minimum temperatures in the first quarter of 2020. For example, Slightly increased rainfall in São Paulo in January and February may have contributed to the higher-than-usual prevalence at the start of the dengue season. Rio de Janeiro also observed higher rainfall and humidity in early 2020 but at the same time had atypically low maximum temperatures, which may have been less conducive to mosquito population growth. In the Northeast, all states experienced some rainfall outliers. In a cluster of states in the extreme Northeast (Rio Grande do Norte, Paraíba, Pernambuco, Alagoas, and Sergipe), these rainfall outliers occurred from March to May. In the remaining states of the Northeast increased rainfall occurred in January and February, accompanied by slightly reduced maximum temperatures, especially in Piauí and Maranhão.

#### South.

**Figure 6:**
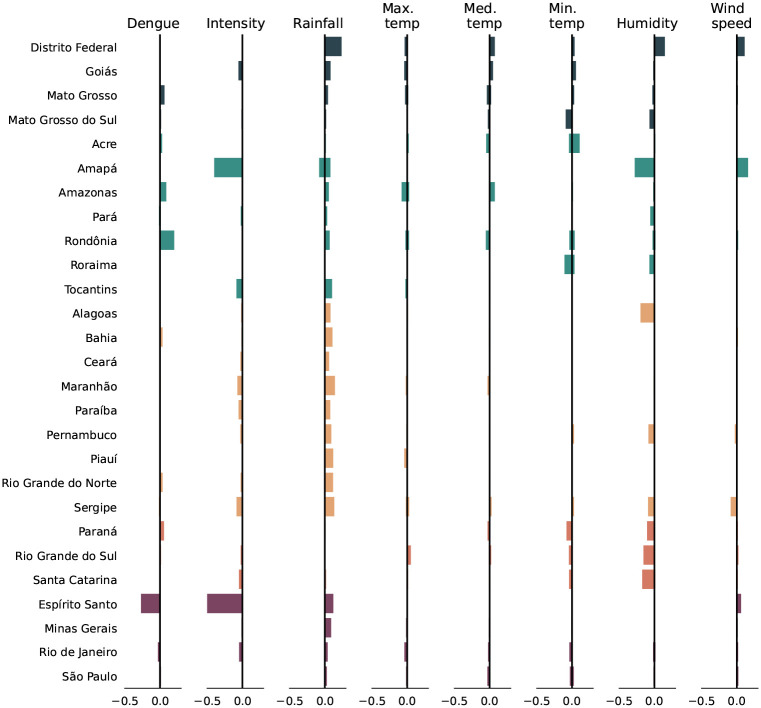
Share of observations with atypically high (positive share) and low (negative share) values by variable and state, colored by region Note: Total observations are all possible week-city combinations for dengue and climate outliers and all possible year-city combinations for intensity outliers.

The southern states experienced unusually dry weather (epidemiological weeks 10–25, March-May) and some colder than usual nights (minimum temperature outliers) (Figure 6). Given that dengue vectors favor humid and warm climates [10], atypical climate conditions alone do not explain the large dengue outbreak in these states.

#### Center-West.

The Center-West federative units, especially Distrito Federal and Goiás, experienced more rainfall than usual in the first quarter of 2020. This may have contributed to the larger-than-expected outbreak size in Distrito Federal.

### 3.4. Role of human susceptibility

Infection with a given dengue serotype confers full immunity against that serotype and temporary protection against other serotypes. Periods of low incidence following large outbreaks have been observed in Brazil and elsewhere, though the periodicity of dengue outbreaks is not yet fully understood [8].

After two years of low dengue prevalence in 2017 and 2018, many regions of Brazil experienced large outbreaks in 2019. This points toward a considerable level of immunity at the start of the COVID-19 pandemic [8].

To approximate population-wide immunity, we compute the sum of dengue infections in the past three years. The year-on-year difference in this measure is negatively correlated with observed dengue cases, suggesting that as immunity declines, observed cases increase. The correlation coefficients are −0.68, −0.51, and −0.15 in 2018, 2019, and 2020, respectively. The relatively weaker relationship in 2020 may suggest the presence of other forces, but more research on longer time series and with other susceptibility measures is needed to verify this interpretation.

**Figure 7:**
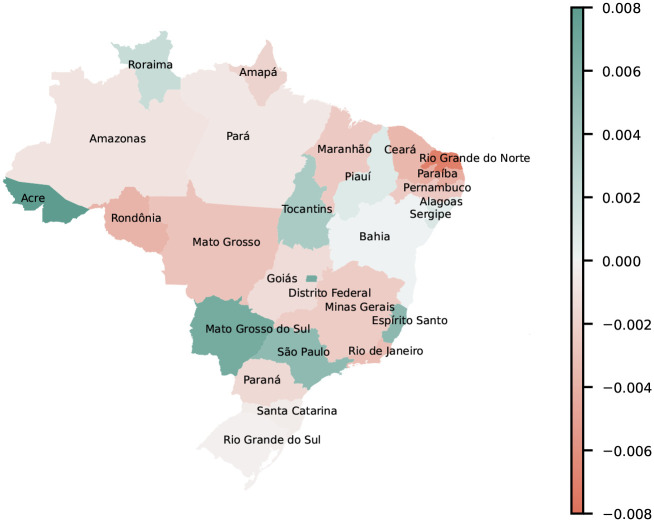
Change in 3-year cumulative dengue incidence, 2019–2020

Figure 7 shows the change in 3-year cumulative per-capita case counts from 2019 to 2020. Positive values in states such as São Paulo, Mato Grosso do Sul, Espírito Santo, and Acre suggest greater immunity, and therefore lower susceptibility. The largest decreases in approximated immunity occur in the Northeastern states Rio Grande do Norte, Paraíba, and Ceará.

The Northeast observed fewer dengue infections than expected, which is not explained by climate factors, but which may be partially due to the decreased immunity in the population. In Paraíba and Rio Grande do Norte, for example, this reduction in susceptibility could help explain some of the remaining gap between expected and observed cases after accounting for excess under-reporting (Figure 4).

### 3.5. Remaining effects: human mobility and over-reporting

The remaining unexplained deviations between observed and expected cases may be in part driven by the two remaining consequences of the COVID-19 pandemic, human mobility and over-reporting. However, these two factors are difficult to assess empirically, due to both data limitations and bidirectional effects. This section considers the available evidence for each of these factors.

#### 3.5.1. Evidence for human mobility impacts

**Figure 8:**
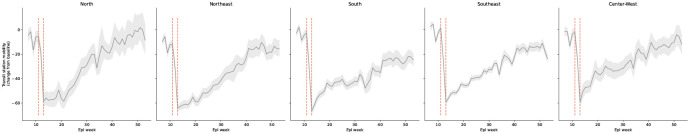
Change in transit station mobility, relative to pre-pandemic baseline Gray lines and shaded area represent the mean and 95% confidence interval of Google mobility index across cities in each region. Orange vertical dotted lines highlight epidemiological weeks 11–13.

In spite of some variability in the timing of non-pharmaceutical interventions, such as school closures, the empirical data from Google users shows that mobility reductions occurred simulta-neously across all of Brazil, starting around epidemiological week 11 (March 8–14) and reaching the lowest point in week 13 (March 22–28). Mobility slowly returned to near-pre-pandemic levels over the course of the year (Figure 8). Given the extrinsic incubation period, we expect impacts on dengue to manifest themselves one to three weeks after the start of mobility reductions and stay in place while mobility remains low.

#### 3.5.2. Evidence for over-reporting

The literature suggests the possibility of over-reporting of dengue cases during the COVID-19 pandemic, due to similarity of symptoms and cross-reactivity of laboratory tests for DENV and SARS-CoV-2 [38, 32, 29]. The historically low dengue case-fatality rate of only 4% in 2020 suggests that over-reporting may have also been a reality in Brazil [37, 36]. However, this effect is difficult to quantify rigorously at the sub-national level with existing data sources.

**Figure 9:**
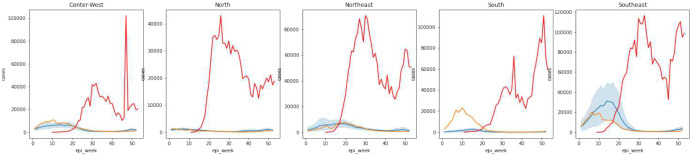
Prevalence of COVID-19 (red) and dengue (2014–19: blue, 2020: orange), by region

Figure 9 shows reported dengue and COVID-19 cases across Brazil’s regions. The relatively late increase in observed COVID-19 cases in the South and Center-West could signal either delayed spread of the disease to these regions or delayed identification of SARS-CoV-2 infections, which would help explain the unusually large dengue case counts observed in these regions.

## 4. Discussion

We analyzed epidemiological, meteorological, and human mobility data to assess the impact of the COVID-19 pandemic on dengue in Brazil. We employed causal inference using an interrupted time series design together with statistical analyses to estimate the respective roles of climate conditions and human susceptibility, as well as changes in dengue surveillance and human mobility during the COVID-19 pandemic. Overall, most states experienced fewer dengue cases than expected and all states had evidence of excess under-reporting. However, there was considerable geographic variation in the pandemic’s overall impact and the role of individual drivers.

States in the North had the lowest discrepancy between observed and expected cases. This finding reflects the fact that the first COVID-19 wave occurred late in the dengue season in the North. Given that disease control interventions are most effective early in a dengue outbreak [10], we would therefore expect this region to experience the least impact of the pandemic. Most states in the Northeast and Southeast registered fewer dengue infections than usual, which is consistent with the evidence on excess under-reporting in these regions. In the Northeast, we observe an especially pronounced dip in case counts in the first two weeks of March. Southern states experienced unusually large dengue outbreaks, which may be explained by a combination of increased human susceptibility and over-reporting, since climate conditions do not warrant the large outbreak size and since the timing of outbreak onset is not consistent with increased dengue spread due to mobility reductions. Similar to the South, states in the Center-West observe large dengue outbreaks early in the season, partially explained by increased rainfall and susceptibility in some states.

However, complex mechanisms guide the impact of the COVID-19 pandemic on dengue in Brazil, and more research and data are needed to gain greater certainty on the exact effect sizes of both pandemic and non-pandemic factors. For example, mechanistic models may help identify the most likely mobility scenarios. Serological studies of neonatal blood spot or blood donor data can provide a groundtruth estimate of true dengue incidence in 2020. Finally, a large-scale genomic analysis could help assess which dengue strains were circulating during the pandemic, estimate the corresponding level of immunity against those strains within the local population, and identify whether there were fewer pathogen introductions when mobility was reduced.

This study has several limitations. Firstly, our causal estimates rely on the quality of our predictive models. Forecasting is an inherently difficult task, especially over long prediction horizons, and further complicated by the threat of time-varying confounders. To mitigate some of the risks, we utilize two distinct model types for the interrupted time series analysis and also perform a separate outlier analysis. Results are qualitatively similar for all models in our study.

Secondly, guaranteeing the fulfilment of the three conditons of causal inference, ignorability, positivity, and consistency, is challenging in non-experimental settings [24]. The pandemic, as it relates to dengue, is a composite intervention, which may violate the consistency assumption of causal inference. We aim to mitigate this limitation by separately considering different aspects of the pandemic.

In addition to model bias, we expect results to be affected by data limitations, since we rely on approximations for the many of the variables of interest. While human susceptibility, for example, is known to affect dengue dynamics, we lack data on population-wide immunity, such as large-scale serological surveys at the start of the pandemic. We consider the year-on-year change in the sum of infections of the three previous years as an approximation of variation in immunity across locations. To limit some of these data biases, we used multiple data sources for the approximations, where feasible. For example, we consider several climate variables and two proxies for excess under-reporting. Further research may consider other methods to estimate dengue covariates and will be an important avenue of investigation to compensate the data gaps created by the COVID-19 pandemic more broadly.

Assessing the 2020 dengue landscape mirrors surveillance challenges in other areas of public health. The SARS-CoV-2 pandemic not only created data gaps, but also altered underlying data generation mechanisms, such as transport patterns, social networks, or trust in public health policies. This is a serious obstacle to monitoring health outcomes and implementing evidence-based policy. This study has contributed to reducing some of these data gaps and increasing our understanding of the causal drivers of dengue spread in Brazil, which is an important precursor to developing effective public health interventions for dengue control in the future.

## Supplementary Material

1
