# Supplementary material for: Estimating the impact of the COVID-19 pandemic on dengue in Brazil
Source: Res Sq. 2023 Feb 9:rs.3.rs-2548491. Preprint. [Version 1] doi: 10.21203/rs.3.rs-2548491/v1 (PMC9934738; doi:10.21203/rs.3.rs-2548491/v1)
Supplement: 1 [file NIHPPRS2548491V1-supplement-1.pdf]

Supplemental Text: Causal model

Observed dengue cases are a consequence of two processes: the disease dynamics underlying the observed and unobserved dengue infections and the associated surveillance systems. Both of these processes may have been impacted by the COVID-19 pandemic, specifically by changes in human mobility and disease reporting as a consequence of altered care-seeking behavior and care availability. Underlying our analysis is the causal model of dengue transmission shown in Fig. 10. The following text describes each of the nodes in the causal model (highlighted in bold face in the text).

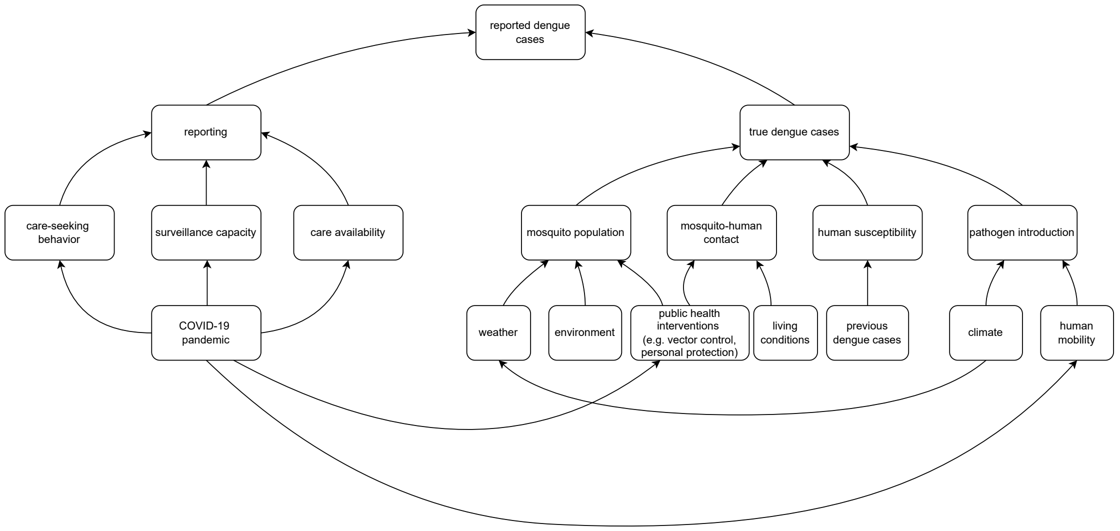

Figure 10: Graphical causal model

5.1. Causes of disease dynamics

Dengue dynamics are driven by a complex interaction of biological, ecological, and social factors of the vector, host, and pathogen. Dengue transmission in humans is mediated by *Aedes aegypti* and *Aedes albopictus* mosquitoes. The **mosquito population** size is affected by environmental (e.g. presence of larval breeding sites) and climate conditions (e.g. temperature, humidity), which define the limits of vector survival. Within these limits, we distinguish between viable and optimal conditions. Campbell and colleagues (2013), for example, find that 80 percent of dengue cases in Thailand occurred when mean humidity was above 75 percent and mean temperature was within 27–29.5°C [10]. This is because the extrinsic incubation period decreases at warmer temperatures (30°C instead of 25°C), thus reducing the time it takes for a mosquito to transmit the virus between humans [12]. Similarly, rainfall and flooding can provide additional breeding habitats for Aedes mosquitoes, for example after extreme weather events such as hurricane Mitch in Central America in 1998 [18, 17]. Dengue is observed in all states in Brazil, though climate zones span tropical and sub-tropical regions and thus differ in the length and strength of viability conditions.

In addition to the mosquito population size, the rate of **human-mosquito contact** is influenced by factors such as the location and type of human dwellings, including the availability of breeding sites near the home, the use of air-conditioning or -filtration, and population density.

Public health interventions and personal protective measures may affect both the mosquito population size and human-mosquito interactions. For example, campaigns urging residents to cover water containers during droughts can help remove mosquito breeding habitats near homes. Redirection of resources and attention to SARS-CoV-2 may have impacted public health interventions.

Upon contact with an infectious mosquito, the probability of infection is determined by a person's immune status. Previous infection with a dengue serotype conveys lifelong immunity against that serotype and temporary, partial immunity against other serotypes. **Human susceptibility** to dengue may thus be reduced after large outbreaks.

Finally, disease spread relies on the **presence of the pathogen**, which may be affected by human mobility, one of the key factors affected by the COVID-19 pandemic. Given the short travel radius of *Aedes mosquitos* of 100-400 meters [49, 34, 31], spread of dengue, especially in non-endemic regions, relies on (re-)introduction of the pathogen by infected humans, who travel across neighborhoods, cities, and countries [28, 44, 26, 47]. Whether increased mobility leads to increased dengue cases is not completely understood, but may vary by geography and may be affected by the variability of mobility [1], or the mosquito density and the biting suitability of humans [41].

5.2. Disease reporting

The observed case count is also impacted by the share of infections that is ascertained. Dengue reporting involves three steps: (i) care-seeking of the patient, (ii) availability of care, and (iii) subsequent reporting upon diagnosis. The pandemic may have affected reporting through all three of these channels. Care-seeking behavior may be affected by physical and financial access to healthcare and willingness to engage with the healthcare system, which may have been altered by fear of COVID-19 infection, transport availability, and economic impacts of the pandemic. Capacity to treat conditions other than COVID-19 were also reduced during the pandemic. Finally, overburdened healthcare systems and repurposing of resources to combat COVID-19 may have reduced the level of disease monitoring [16, 2, 35, 46, 6].

Dengue is a nationally notifiable disease and healthcare providers are required to report suspected cases if a patient in an area of dengue transmission or *Aedes aegypti* infestation presents with fever and at least two of the following symptoms: headache, retro-orbital pain, myalgia, arthralgia, exanthema, bleeding or hemorrhage, nausea or vomiting, petechiae orpositive tourniquet test, or leukopenia [33, 43]. Given the overlap with common symptoms of COVID-19, including fever, headache, nausea or vomiting, and myalgia, symptom-based misreporting of the two diseases in the early pandemic period is plausible. This hypothesis is strengthened by the fact that cross-reactivity of lab tests for both DENV and SARS-CoV-2 infections has been reported [38, 32, 29].

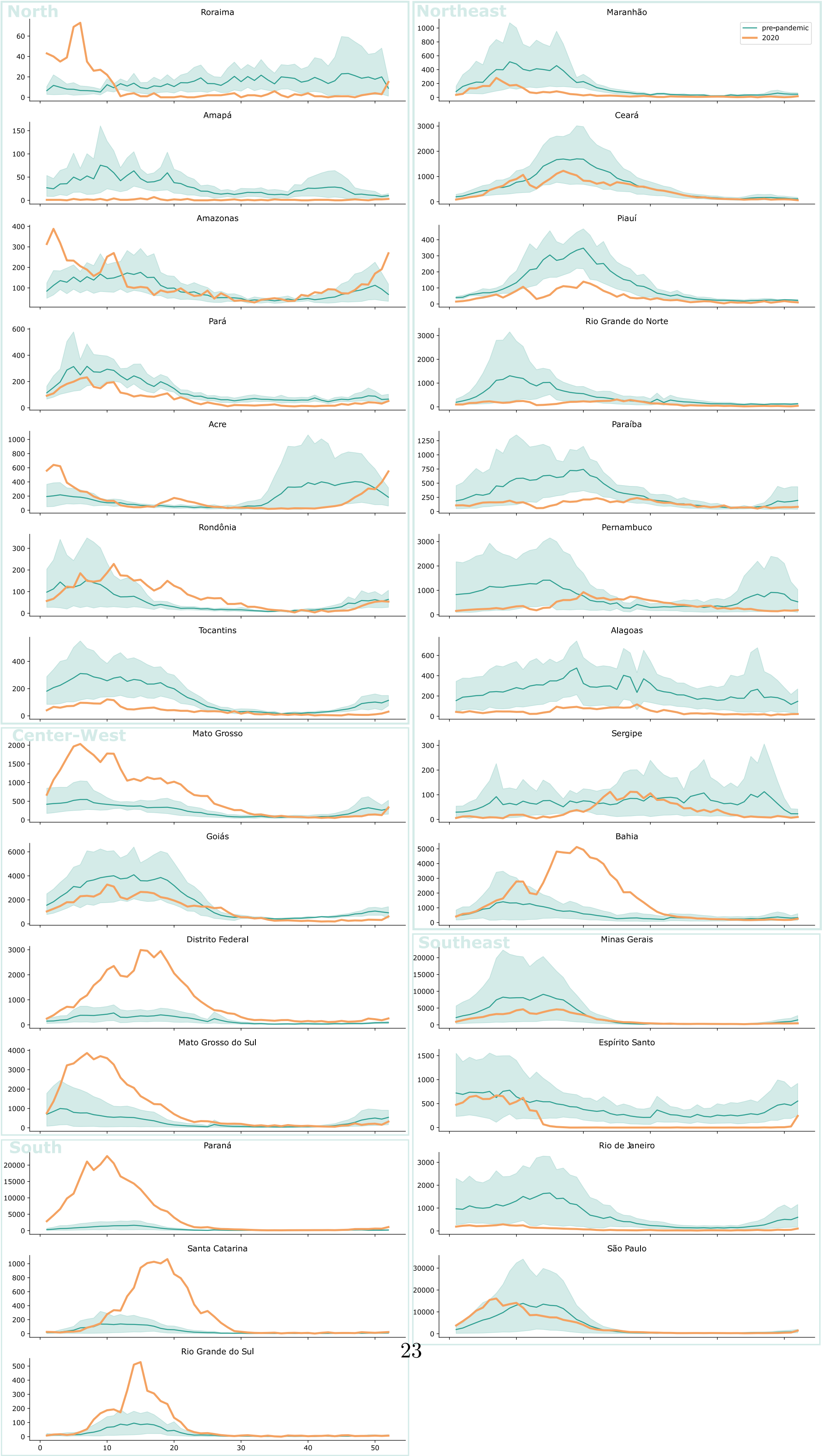

Figure 11: Dengue time series by epidemiological week in 2020 and in the pre-pandemic period 2014-19 (mean and 95% confidence interval), by state and region

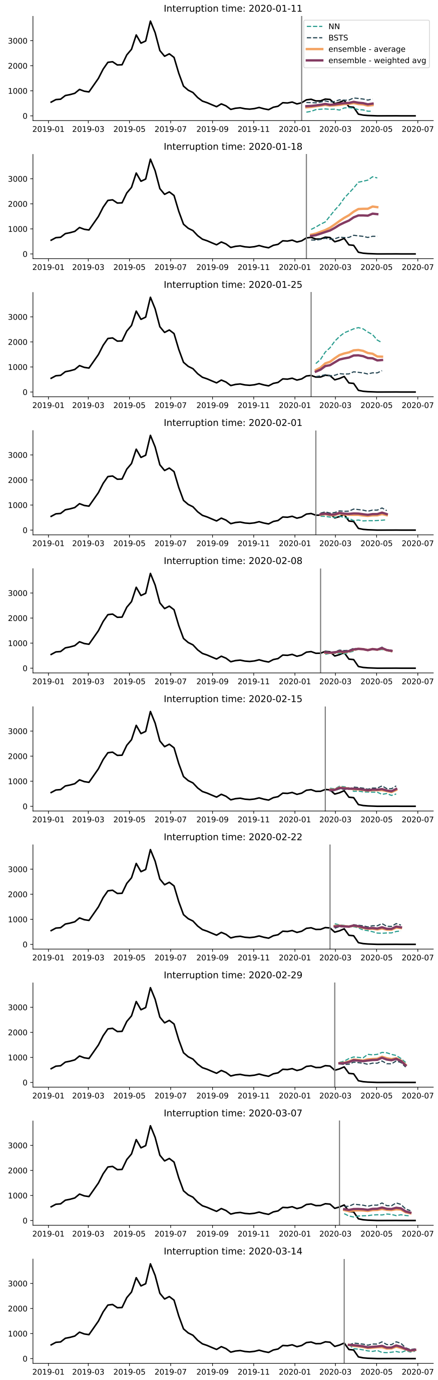

Figure 12: Interrupted time series analysis forecasts for Espírito Santo using different predictive models at different interruption times

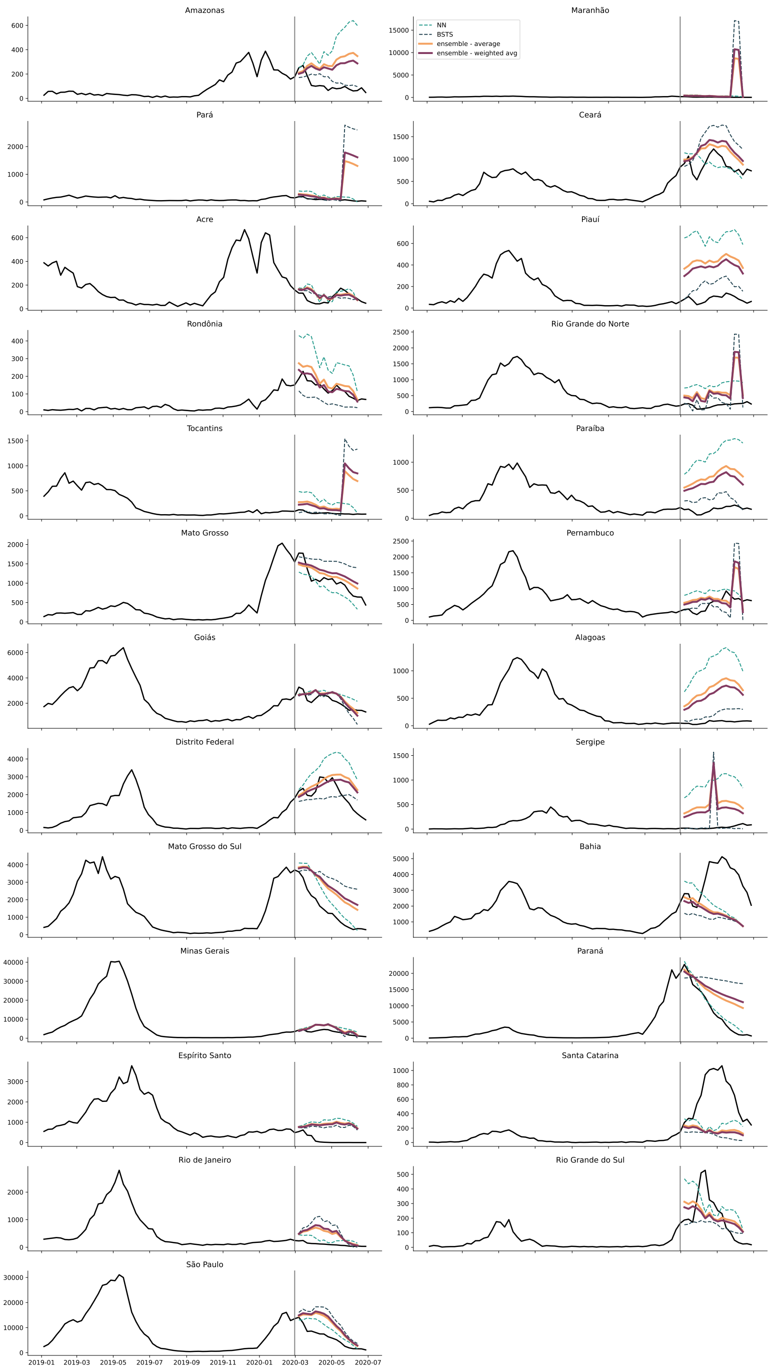

Figure 13: Predicted and observed dengue cases in all states for interruption point Feb 29th

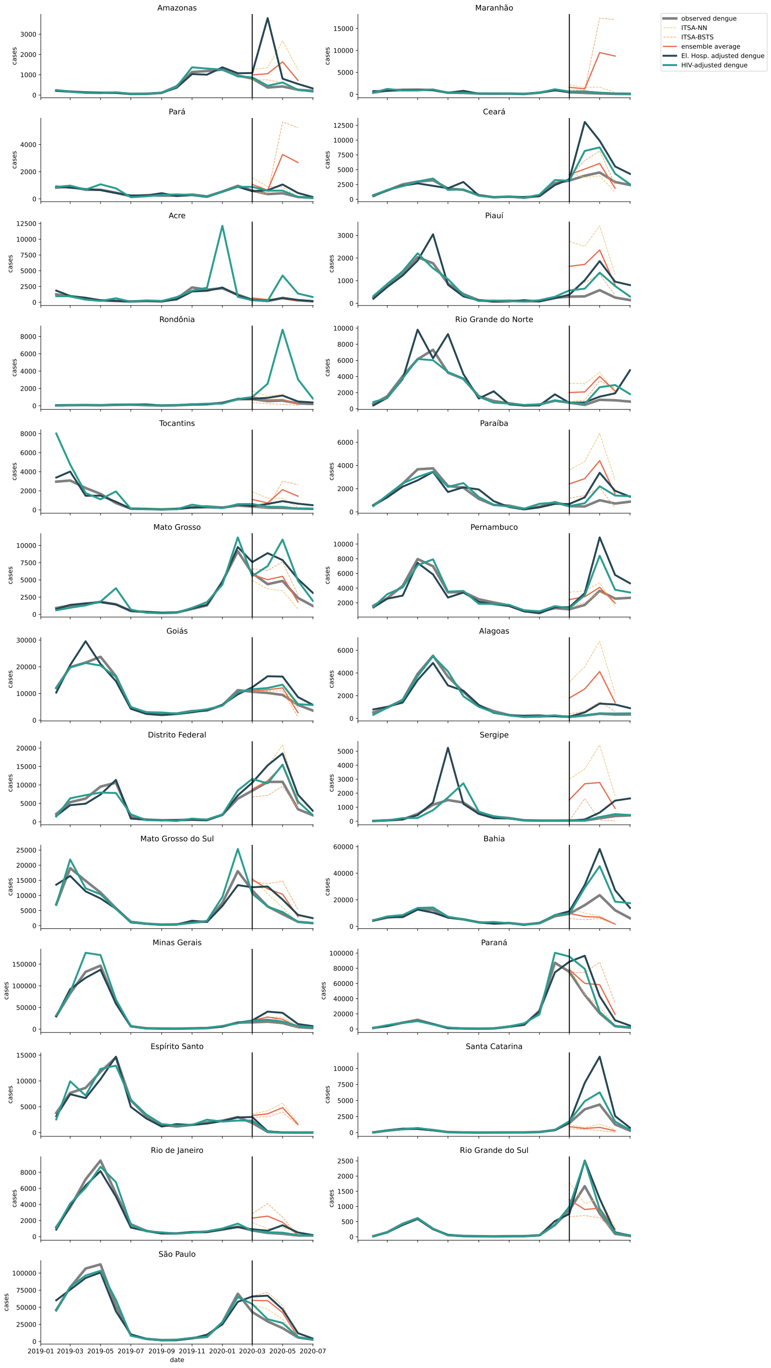

Figure 14: Comparing monthly reporting-adjusted dengue cases with observed and expected dengue cases for all states at interruption time March 1st
